# Efficacy of Wingman Crossing Catheter to Penetrate a Chronic Total Occluded Ostium Without Stump in a Superficial Femoral Artery

**DOI:** 10.1002/ccr3.71170

**Published:** 2025-10-06

**Authors:** Eiji Karashima, Takeo Kaneko

**Affiliations:** ^1^ Department of Cardiology Shimonoseki City Hospital Yamaguchi Japan

**Keywords:** endovascular treatment, superficial femoral artery, Wingman chronic total occlusion

## Abstract

Here, we report a case of endovascular treatment in which the Wingman crossing catheter evaluated the efficacy of penetrating a chronic total occluded ostium without a stump in a superficial femoral artery.

## 1 Case Presentation

Penetrating an ostium of a chronic total occlusive (CTO) lesion without a stump in a superficial femoral artery (SFA) is technically challenging and time‐consuming. The Wingman crossing catheter (ReFlow Medical Inc., San Clemente, CA, US) is a CTO‐crossing catheter with a blade tip for CTO penetration. The efficacy and safety of this catheter for crossing the SFA‐CTO were reported [1]. In this case, we used a Wingman catheter to penetrate the ostium of SFA‐CTO without a stump.

**FIGURE 1:**
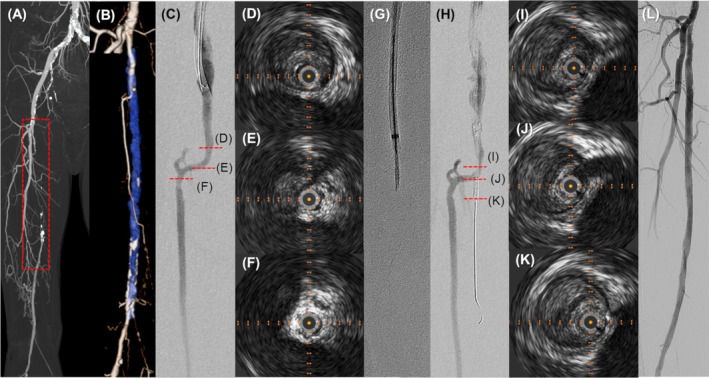
Images of right femoropopliteal artery. (A) CT angiography. (B) Magnified CT angiography with a virtual occlusive vessel image of the area in the rectangle in panel (A). (C–F) Angiography and IVUS findings before using a Wingman catheter. (G) Using a Wingman catheter with a GOGO catheter D‐shape. (H–K) Angiography and IVUS findings after using a Wingman catheter. (L) Angiography after implanting two drug‐eluting stents.

A 64‐year‐old male with claudication that had not improved with the guideline‐directed medical therapy and exercise therapy underwent an endovascular treatment. Computed tomography (CT) angiography showed right SFA occlusion (Figure 1A,B). At the ostium of SFA‐CTO, angiography and intravascular ultrasound (IVUS) showed a branch artery but failed to detect the blood flow into the SFA (Figure 1C–F). Because the wires could not penetrate the ostium of SFA‐CTO with IVUS‐guided and extravascular ultrasound‐guided wiring methods, we used a Wingman 18 catheter (Figure 1G). If using the Wingman catheter did not work, we planned to perform retrograde wiring, a more economical method. After penetrating the ostium of SFA‐CTO with a Wingman catheter, wires could be crossed into the SFA‐CTO. Intraplaque wiring without any vessel perforation was detected by angiography and IVUS (Figure 1H–K). Severe dissection was observed after the balloon angioplasty; two drug‐eluting stents were implanted in the SFA with no periprocedural complications (Figure 1L).

It should be noted that the use of a Wingman catheter might increase the risk of vessel perforation because it is difficult to control the direction of the blade tip at will [2]. We used a GOGO catheter D‐shape (Medikit, Tokyo, Japan) to avoid vessel perforation and to direct the blade tip toward the intraplaque as much as possible (Figure 1G). In this case, using a Wingman catheter was a simple, useful, and time‐saving method that avoided distal puncture to penetrate the SFA‐CTO ostium without a stump.

## Author Contributions

**Eiji Karashima:** conceptualization, data curation, writing – original draft. **Takeo Kaneko:** writing – review and editing.

## Ethics Statement

The authors have nothing to report.

## Consent

Written informed consent was obtained from the patient to publish this report in accordance with the journal's patient consent policy.

## Conflicts of Interest

The authors declare no conflicts of interest.

## Data Availability

All data are included in the case report.
